# Effects of Glutamine or Glucose Deprivation on Inflammation and Tight Junction Disruption in Yak Rumen Epithelial Cells

**DOI:** 10.3390/ani14223232

**Published:** 2024-11-12

**Authors:** Ziqi Yue, Junmei Wang, Rui Hu, Quanhui Peng, Hongrui Guo, Huawei Zou, Jianxin Xiao, Yahui Jiang, Zhisheng Wang

**Affiliations:** 1 Low Carbon Breeding Cattle and Safety Production University Key Laboratory of Sichuan Province, Sichuan Agricultural University, Chengdu 611130, China; 2 Key Laboratory of Animal Diseases and Environmental Hazards of Sichuan Province, Sichuan Agricultural University, Chengdu 611130, China

**Keywords:** yak rumen epithelial cells, glutamine deprivation, glucose deprivation, inflammation, tight junction

## Abstract

Yak is a special free-ranging cattle breed in the plateau areas of Qinghai and Tibet. Pasture withering in cold-season pastures results in energy deficiency in yaks, which undermines the rumen epithelial barrier. However, the leading factor causing rumen epithelial injury remains unknown. Glutamine (Gln), a conditionally essential amino acid, is insufficient under pathological conditions. Glucose (GLU) is an important energy source. Thus, we explored the effects of Gln or GLU deprivation on the barrier function of yak rumen epithelial cells and investigated the underlying mechanisms, as well as the differences in rumen epithelial barrier function between Gln deprivation (Gln-D) and GLU deprivation (GLU-D). In previous work, we constructed the yak rumen epithelial cells (YRECs) line by transferring the human telomerase reverse transcriptase gene (hTERT) and simian virus 40 large T antigen (SV40T) into primary YRECs. The YRECs were exposed to normal, Gln-D, GLU-D, and serum replacement (SR) media for 6, 12, and 24 h. Our data displayed that cell viability and tight junction protein expression in the SR group were not significantly changed compared to the normal group. Whereas, compared with the SR group, Gln-D treated for more than 12 h reduced cell viability and proliferation, and GLU-D treated for more than 12 h damaged the cell morphology and reduced cell viability and proliferation. The cell proliferation and cell viability were decreased more in GLU-D than in Gln-D. In addition, Gln-D treated for more than 12 h disrupted YREC cellular partially tight junctions by inducing oxidative stress and inflammation, and GLU-D treated for more than 12 h disrupted YREC cellular tight junctions by inducing apoptosis, oxidative stress, and inflammation. Compared with Gln-D, GLU-D more significantly induced cell injury and reduced tight junction protein levels. Our results provided evidence that GLU-D induced damage through the p38 mitogen-activated protein kinase (p38 MAPK)/c-junN-terminal kinase (JNK) signaling pathway, which was more serious than Gln-D treated for more than 12 h.

## Simple Summary

Nutrient deficiency in yak has become a common phenomenon because of pasture deficiency in the cold season in the plateau regions. In ruminants, the rumen plays an important function in the digestion and absorption of nutrients. The previous study of our research group reported that forage deficiency significantly decreased the concentration of glutamine (Gln), thus leading to the inflammation and barrier function damage of yak ruminal epithelium tissue in the cold season, which inhibited yak growth. However, the specific molecular mechanisms are still unclear. Thus, we explored the mechanism of the effects and the differences between the Gln and glucose (an important energy source, GLU) deprivation on epithelial cell function. The results showed that Gln deprivation (Gln-D) beyond 12 h disrupted YREC cellular tight junctions by inducing oxidative stress and glucose deprivation (GLU-D) beyond 12 h disrupted yak rumen epithelial cell (YRECs) cellular tight junctions by inducing apoptosis and oxidative stress. Meanwhile, Gln-D or GLU-D significantly improved the p38 mitogen-activated protein kinase and JNK protein expression levels. Consequently, the harmful effect of Gln-D or GLU-D might be associated with the activation of both the p38 MAPK and JNK signaling pathways in YRECs. Also, GLU-D induced injury more serious to YRECs than Gln-D. Furthermore, GLU-D reduced the levels of tight junction gene expression in YRECs compared with Gln-D.

## 1. Introduction

Yaks (*Bos grunniens*) are grazed on the Qinghai-Tibetan Plateau all year and face severe challenges in the cold season, including limited food availability and extreme cold [1,2]. Thus, the nutrient deficiency of grazing yaks caused by the scarcity of forage has become a ubiquitous phenomenon, which may limit the healthy growth of yaks [3]. Severe starvation or nutrient deficiency inhibited gastrointestinal development, resulting in poor growth performance in yaks [4]. It was reported that nutrient deficiency reduced the apparent digestibility of crude fat, crude protein, and acid-detergent fiber in yaks, which decreased the weight [5].

Rumen is a unique organ in ruminant animals. There were many researchers showed the function of rumen in digestion, metabolism, nutrition absorption, and immune barrier function [1,6,7]. The ruminal epithelium is an important site of digestion for ruminants, as it helps absorb and transport nutrients, as well as form a barrier to endotoxins originating from the ruminant and its feed [8]. The previous study of our research group reported that forage deficiency induced inflammation and barrier function damage in yak ruminal epithelium tissue in the cold season, which resulted in growth-retarded yaks [4]. The epithelial barrier integrity largely relies on the steady state of healthy epithelial cells, mainly including immune responses, tight junction integrity, cell proliferation, and programmed death [9]. The decreased forage intake caused inadequate energy intake in the rumen epithelium. In addition, Glutamine (Gln), a conditionally essential amino acid, is insufficient under pathological conditions [10]. Studies have found that energy or Gln deficiency induces cell damage [11,12,13]. In mammalian cells, Gln is now well-established as an alternative energy to glucose (GLU) for adenosine triphosphate production and biosynthesis [14].

Living cells utilize GLU as an energy source [15]. Gln is the most plentiful and versatile amino acid in the animal body, which is not only an important energy substance for cells but can also be used as a signaling substance to regulate a variety of cellular physiological functions [10]. Therefore, Gln and GLU are common limiting substrates during cell cultivation [16]. As reported, Gln and GLU deprivation-induced oxidative damage by increasing reactive oxygen species (ROS) levels in MCF7 cells and PDA cells, respectively [11,17]. Furthermore, GLU deprivation up-regulated *IL-6* mRNA expression in adipose-derived stromal/stem cells (ASC) [12]. The mitogen-activated protein kinase (MAPK) signaling pathway has been identified as a crucial pathway that regulates a wide range of cellular functions, such as apoptosis, oxidative stress, cell proliferation, inflammation, and tight junction protein expression [13,18]. A study found that Gln deprivation reduced the mRNA levels of pro-inflammatory cytokines in U2OS cells via c-junN-terminal kinase (JNK) signaling [19]. Another study showed that GLU deprivation induced apoptosis in N2A cells via p38 MAPK signaling [20]. Therefore, we speculated that Gln and GLU deprivation might impair yak rumen epithelial cells through the MAPK signaling pathway.

Inflammation and epithelial tight junction impairment in the gastrointestinal tract induced by pasture withering in cold-season pastures are the crucial reasons for growth-retarded yaks [4]. Inadequate forage intake results in energy and Gln deficiency in the rumen epithelium. However, the leading factor causing rumen epithelial injury remains unknown. Thus, in this study, we used the yak rumen epithelial cells (YRECs) as a model and investigated the possible mechanisms and differences between Gln or GLU deprivation on tight junction injury and inflammatory response. In parallel, the research might provide a theoretical basis for studying the damage of forage deficiency on yak rumen epithelium in the cold season.

## 2. Materials and Methods

### 2.1. Chemicals and Reagents

Roswell Park Memorial Institute (RPMI) 1640 medium, Glutamine-free and glucose-free RPMI 1640 medium, serum replacement (SR), and Fetal bovine serum (FBS) were bought from Gibco (Shanghai, China). Penicillin-streptomycin-amphotericin, the Bicinchoninic acid (BCA) kit, and 4% paraformaldehyde were bought from Solarbio (Beijing, China). Protease inhibitors and phosphatase inhibitors, 5 × loading buffer, hematoxylin–eosin (HE) staining kit, CCK-8 kit, 5-ethynyl-2′-deoxyuridine (EDU) Cell Proliferation Assay Kit, and 0.25% trypsin were purchased from Beyotime (Shanghai, China). l-Methionine-dl-sulfoximine was bought from MCE (MSO) (Princeton, NJ, USA). Supplementary Table S1 contains the list of antibodies. Six-well and 96-well cell culture plates were bought from NEST (Wuxi, China). Selleck (Houston, TX, USA) provided the inhibitor of JNK (SP600125) and p38 MAPK (SB203580). The glutathione peroxidase (GSH-PX), total antioxidant capacity (T-AOC), malondialdehyde (MDA), and superoxide dismutase (SOD) assay kit were bought by Nanjing Jiancheng Bioengineering Institute (Nanjing, China). SteadyPure Quick RNA Extraction kit was bought from Accurate Biology (Changsha, China). SYBR Green q-PCR Master Mix kit was bought from Servicebio (Wuhan, China). Exongen (Chengdu, China) provided the reverse transcription kit.

### 2.2. Cell Culture and Experiment Design

The YREC line was established by our laboratory. Briefly, the tissue block culture, enzyme digestion, and tissue block digestion method were used to isolate primary YRECs, and we constructed the YRECs line by transferring the human telomerase reverse transcriptase gene (hTERT) and simian virus 40 large T antigen (SV40T) into primary YRECs. Meanwhile, our research revealed that YRECs could form tight polarized monolayers, the transepithelial electrical resistance (TEER) value reached 759.36 Ω·cm^2^, and the transmittance of fluorescein isothiocyanate dextran was below 1% [21].

The YRECs were cultured in RPMI 1640 medium (2 mM Glutamine and 2 mM Glucose) with 1% penicillin-streptomycin-amphotericin and 10% FBS in a 5% CO_2_ incubator at 37 °C. YRECs in passages 20–30 were used for the experiment in our study. As soon as the YRECs reached 80–90% confluence, the medium was replaced with fresh complete medium (RPMI 1640 medium and serum, CON), glutamine deprivation medium (Glutamine free RPMI 1640 medium and serum replacement, 4 mM MSO, Gln-D), glucose deprivation medium (Glucose free RPMI 1640 medium and serum replacement, GLU-D), and serum replacement medium (RPMI 1640 medium and serum replacement, SR). There was no glucose or glutamine in the SR. The SR was added to both the Gln-D and GLU-D groups. The SR group was created to rule out the effects of serum replacement on the results. The YRECs were treated for 6, 12, and 24 h in the present study.

### 2.3. Cell Morphology

To explore the influence of GLU-D and Gln-D on cell morphology, the cells in different treatment groups were stained by HE staining. In brief, the medium was changed to the different treatment mediums (CON, Gln-D, GLU-D, and SR) as soon as the cells reached 80–90% confluence. After 6, 12, and 24 h of treatments, we fixed the cells with 4% paraformaldehyde, then stained with HE.

### 2.4. Cell Viability

A CCK-8 kit was used to measure the YRECs viability. Briefly, in the 96 well plates, 10 μL of CCK-8 solution was poured into each well, the solution and the cells were co-incubated at 37 °C for 1 h, and the OD value of cells was determined at 450 nm using a microplate reader.

### 2.5. Cell Proliferation

Based on instructions provided by the manufacturer, EDU assays were executed using the EDU Cell Proliferation Assay Kit. In brief, cell culture plates were added with EDU solution and incubated at 37 °C, 5% CO_2_ incubator, for 1 h. Overall, 4% paraformaldehyde was used to fix the cells, and 0.2% Triton X-100 was used to permeabilize the cells. Then, at room temperature, cells were protected from light, soaked in the click reaction mixture from the test kit for 1.5 h, and counterstained with DAPI for 10 min. Finally, photographs were taken with a fluorescence microscope (Leica, Wetzlar, Germany).

### 2.6. ROS Detection

For ROS detection, YRECs grew to 80–90% in the 6-well plates, then treated with Gln-D, GLU-D, and SR and cultured for 24 h. A flow cytometer was used to detect the ROS level after incubating the cells with 10 µM DCFH-DA solution for 20 min at 37 °C, and the data analysis was performed using FlowJo software (FlowJo LLC, Ashland, OR, USA, FlowJo V 10.8.1).

### 2.7. Antioxidant Capacity Assay

Antioxidant capacity assay including the enzyme activity of GSH-PX, T-AOC, MDA, and SOD. Following the descriptions from the assay kit, the cell lysate was collected and detected immediately. In brief, YREC cell lysate was mixed with kit reagent, then a microplate reader was used to take the OD measurement.

### 2.8. RNA Extraction and Reverse-Transcription Quantitative PCR (RT-qPCR)

The total RNA was extracted according to the manufacturer instructions provided with the SteadyPure Quick RNA Extraction kit. A Reverse Transcription Kit was used to reverse transcribe RNA to cDNA. q-PCR was executed by using the Universal Blue SYBR Green q-PCR Master Mix kit on QuantStudio 5 real-time q-PCR system (Foster city, CA, USA). GAPDH was chosen as the internal control, and three replicates were used for the qPCR. In Supplementary Table S2, the primer sequences for quantitation of target genes are illustrated. Analysis of real-time PCR data was performed using the 2^−ΔΔCT^ method.

### 2.9. Western Blot

We collected cells at the indicated times following treatments in 6-well plates. Cell lysates were prepared using a RIPA-mixed buffer containing protease inhibitors and phosphatase inhibitors. Supernatants were collected after cells were lysed and centrifuged. The protein concentration in different groups was determined using a BCA kit. To denature the proteins, a 5× loading buffer was added to the supernatants, and the mixture was heated at 100 °C for 10 min. Using SDS-polyacrylamide gel electrophoresis, the protein extract was subjected to Western blot analysis. Using primary antibodies overnight at 4 °C, secondary antibodies were applied at room temperature for 1.5 h. We visualized the proteins using a chemiluminescence system and ECL. A quantitative analysis of the Western blot results used ImageJ (V 1.8.0.112). In this study, the antibody information was given in Supplementary Table S1.

### 2.10. Immunofluorescence (IF)

In order to detect the localization of ZO-1 protein in YRECs, immunofluorescence technology was used for detection. The treated cells were fixed with 4% paraformaldehyde for 20 min, next closed with 5% bovine serum albumin (BSA) for 30 min, after incubating with ZO-1 antibody (A0659, ABclonal, Wuhan, China, 1:100) (overnight at 4 °C), then incubated with secondary antibody (room temperature for 2 h), and finally stained with DAPI (room temperature, 5 min). The inverted fluorescence microscope (Leica, Wetzlar, Germany) was used to observe and photograph, and the ImageJ software was used for statistical analysis.

### 2.11. Statistical Analysis

The results of all experiments were analyzed by one-way analysis of variance (ANOVA) followed by Tukey’s HSD post-hoc test (SPSS 27). We used GraphPad Prism 8.0 software to create all of the graphs. The data were expressed as mean ± standard deviation (mean ± SD). Statistical significance was defined as *p* < 0.05.

## 3. Results

### 3.1. Gln-D or GLU-D Impaired the Cell Morphology and Cell Number in YRECs

**Figure 1:**
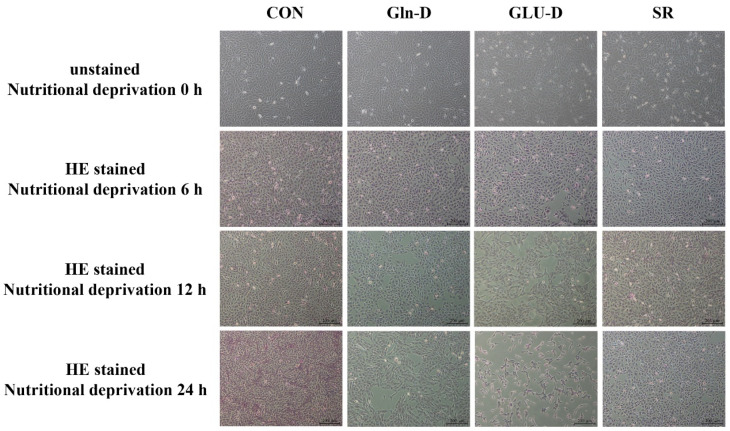
Effects of Gln-D or GLU-D on cell morphology in YRECs at 6 h, 12 h, and 24 h. Cell morphology was observed by HE staining after Gln-D or GLU-D, and the images were taken under a microscope (100×). Scale bars represent 200 μm. HE = hematoxylin–eosin. CON, control; Gln-D, glutamine deprivation; GLU-D, glucose deprivation; SR, serum replacement.

In order to determine the consequence of Gln-D or GLU-D on cell morphology and cell number, YRECs were stained by HE. As shown in Figure 1, compared with the control group, there was no visible change in cell morphology in the Gln-D and SR groups at three time points, while the cell number in the Gln-D group at 12 and 24 h and in the SR group at 24 h was reduced. In the GLU-D group, the cell number was reduced at 12 and 24 h. The cell morphology was not significantly altered after 6 h, but cells appeared shrunken at 12 h, and more shrunken at 24 h.

### 3.2. Gln-D or GLU-D Decreased Cell Viability and Cell Proliferation in YRECs

**Figure 2:**
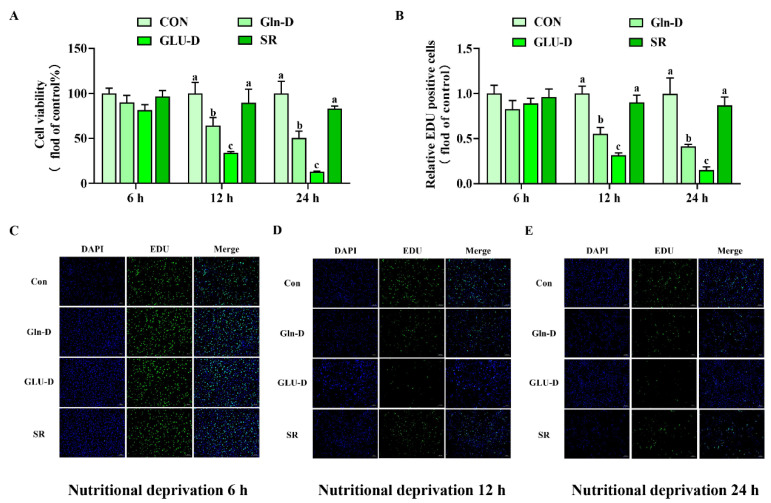
Effects of Gln-D or GLU-D on cell viability and proliferation in YRECs at 6 h, 12 h, and 24 h. (**A**) Cell viability of YRECs assayed by CCK-8. (**B**) EDU-positive cells were detected by Image J. (**C**–**E**) Cell proliferation of YRECs assayed by EDU, and the images were taken under a fluorescence microscope (100×). Scale bars represent 100 μm. Data, expressed as the rate of control cells at each time point, were expressed as means ± SD, n = 3 independent experiments. Different letters indicate significant differences (*p* < 0.05). CON, control; Gln-D, glutamine deprivation; GLU-D, glucose deprivation; SR, serum replacement. EDU = 5-ethynyl-2′-deoxyuridine.

To explore the effect of Gln-D or GLU-D on YRECs cell viability and cell proliferation, we used the CCK-8 kit and EDU staining to detect them, respectively. In Figure 2A–E, SR did not change cell viability and proliferation at three time points compared with the control group (*p* > 0.05). Gln-D or GLU-D groups had no significant effect on cell viability and proliferation at 6 h compared to SR (*p* > 0.05), but significantly inhibited cell viability and proliferation after treatment 12 h and 24 h, respectively (*p* < 0.05). In addition, compared with the Gln-D group, the GLU-D group significantly decreased cell viability and cell proliferation at 12 and 24 h (*p* < 0.05). Overall, these results showed that Gln-D or GLU-D reduced cell viability and cell proliferation in YRECs after treatment for 12 h and 24 h. Moreover, cell viability and cell proliferation were significantly lower in the GLU-D group than in the Gln-D group.

### 3.3. Gln-D or GLU-D Induced Apoptosis in YRECs

**Figure 3:**
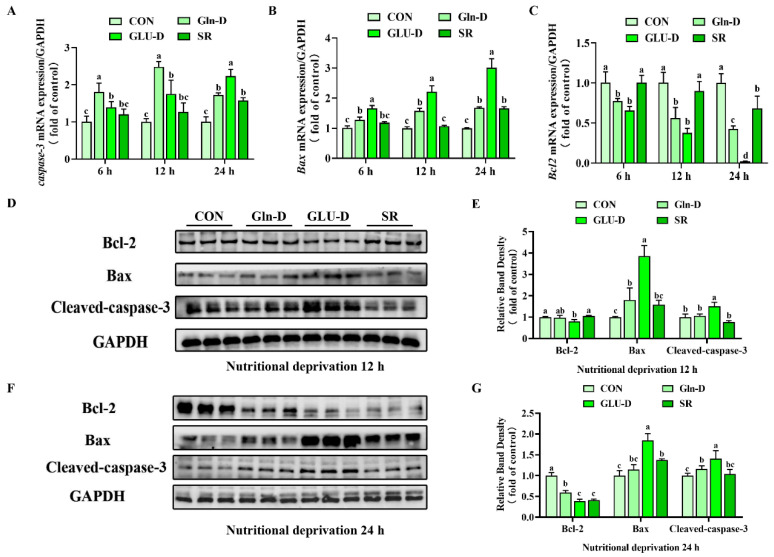
Effects of Gln-D or GLU-D on apoptosis in YRECs. (**A**–**C**) The mRNA levels of *caspase-3*, *Bax*, and *Bcl-2* at 6 h, 12 h, and 24 h. (**D**,**E**) The protein levels of Bcl-2, Bax, and Cleaved-caspase-3, GAPDH were used as a loading control at 12 h. (**F**,**G**) The protein levels of Bcl-2, Bax, and Cleaved-caspase-3 GAPDH were used as a loading control at 24 h. Data, expressed as the rate of control cells at each time point, were expressed as means ± SD, n = 3 independent experiment. Different letters indicate significant differences (*p* < 0.05). CON, control; Gln-D, glutamine deprivation; GLU-D, glucose deprivation; SR, serum replacement. Bax = B-cell lymphoma 2-associated X protein, Bcl-2 = B-cell lymphoma 2, GAPDH = glyceraldehyde-3-phosphatedehydrogenase.

To investigate whether nutrient deprivation affects YRECs apoptosis, we determined the apoptosis-related mRNA and protein levels. As shown in Figure 3A–C, SR did not change the mRNA expression of *caspase-3*, *B-cell lymphoma 2* (*Bcl-2*), and *B-cell lymphoma 2-associated X protein* (*Bax*) at 6 and 12 h compared to the control group (*p* > 0.05). Nevertheless, the transcript levels of *caspase-3* and *Bax* were significantly increased, and *Bcl-2* was significantly decreased at 24 h (*p* < 0.05). Compared with the SR, Gln-D significantly increased *caspase-3* and *Bax* transcript levels but decreased *Bcl-2* transcript levels at 6 and 12 h (*p* < 0.05), GLU-D significantly increased *Bax* transcript levels, while it decreased *Bcl-2* transcript levels at 6, 12, and 24 h (*p* < 0.05) and significantly increased *caspase-3* transcript levels at 24 h (*p* < 0.05). GLU-D significantly decreased *caspase-3* transcript levels but significantly increased *Bax* transcript levels at 6 and 12 h compared to Gln-D (*p* < 0.05). GLU-D significantly increased *caspase-3* and *Bax* transcript levels, while it significantly decreased *Bcl-2* transcript levels at 24 h (*p* < 0.05). Consistently, as shown in Figure 3D–G, SR significantly decreased the Bcl-2 protein level, but increased the Bax protein level at 24 h compared to the control group (*p* < 0.05). GLU-D treated cells had higher protein levels of Bax and cleaved-caspase-3 at 12 and 24 h compared to the SR group (*p* < 0.05). In addition, the protein levels of Bax and Cleaved-caspase-3 significantly increased in the GLU-D group compared to the Gln-D group at 12 h and 24 h (*p* < 0.05). Thus, GLU-D induced YRECs apoptosis at 24 h.

### 3.4. Gln-D or GLU-D Induced Oxidative Stress in YRECs

**Figure 4:**
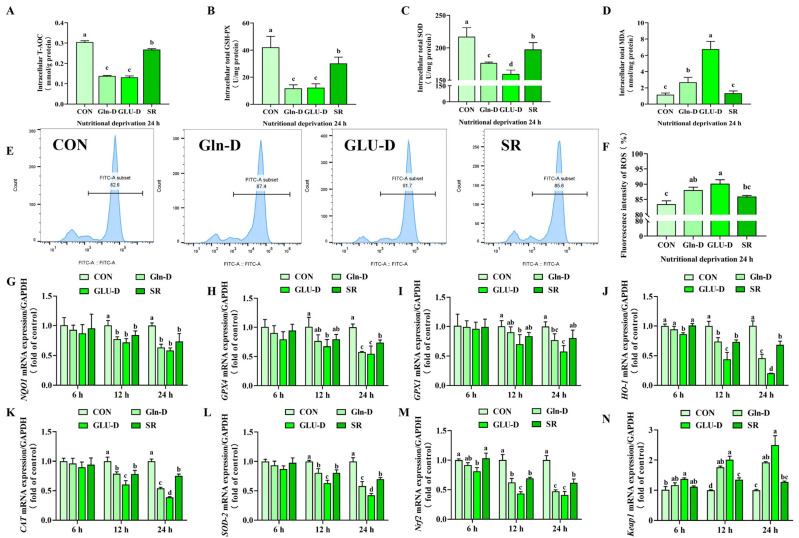
Effects of Gln-D or GLU-D on oxidative stress in YRECs at 6 h, 12 h, and 24 h. (**A**) T-AOC level. (**B**) GSH-PX concentration. (**C**) SOD concentration. (**D**) MDA concentration. (**E**,**F**) The ROS level in YRECs at 24 h was tested by flow cytometry and analyzed by FlowJo software. (**G**–**N**) The mRNA levels of *NQO1*, *GPX4*, *GPX1*, *HO-1*, *CAT*, *SOD-2*, *Nrf2*, and *Keap1*. Data, expressed as the rate of control cells at each time point, were expressed as means ± SD, n = 3 independent experiments. Different letters indicate significant differences (*p* < 0.05). CON, control; Gln-D, glutamine deprivation; GLU-D, glucose deprivation; SR, serum replacement. NQO1 = NAD(P)H Dehydrogenase Quinone 1; GPX4 = glutathione peroxidase 4; GPX1 = glutathione peroxidase 1; HO-1 = heme oxygenase 1; CAT = catalase; SOD2 = superoxide dismutase 2; Nrf2 = nuclear factor-erythroid 2-related factor 2; Keap1 = Kelch-like-ECH-associated protein 1; ROS = reactive oxygen species; T-AOC = total antioxidant capacity; GSH-PX = glutathione peroxidase; SOD = superoxide dismutase; MDA = malondialdehyde.

The next step was to examine whether nutrient deprivation induced oxidative stress in YRECs. We measured the enzymatic activity of GSH-PX, SOD, T-AOC, and MDA by using biochemical kits. The intracellular ROS levels were measured by using flow cytometry and the mRNA levels of *NAD(P)H Dehydrogenase Quinone 1* (*NQO1*), *glutathione peroxidase 4* (*GPX4*), *glutathione peroxidase 1* (*GPX1*), *superoxide dismutase 2* (*SOD2*), *catalase* (*CAT*), *heme oxygenase 1* (*HO-1*), *nuclear factor-erythroid 2-related factor 2* (*Nrf2*) and *Kelch-like-ECH-associated protein 1* (*Keap1*) by q-PCR. As shown in Figure 4A–D, after 24 h treatment, SR significantly decreased the activities of intracellular T-AOC, SOD, and GSH-PX (*p* < 0.05). Gln-D or GLU-D significantly decreased the activities of intracellular T-AOC, GSH-PX, and SOD and significantly increased the activity of MDA compared to the SR group (*p* < 0.05). Furthermore, compared with Gln-D, GLU-D significantly reduced the activity of SOD and significantly improved the concentration of MDA (*p* < 0.05). Meanwhile, GLU-D significantly increased the intracellular ROS levels at 24 h compared to SR (*p* < 0.05) (Figure 4E,F). Furthermore, in Figure 4G–N, SR significantly decreased the transcript levels of *NQO1*, *HO-1*, *CAT*, *SOD2*, and *Nrf2* at 12 and 24 h compared to the control (*p* < 0.05). Compared with the SR, Gln-D significantly increased the *Keap1* transcript level at 12 h, while decreasing the transcript levels of *GPX4*, *HO-1*, *CAT*, *SOD2*, and *Nrf2* at 24 h (*p* < 0.05). GLU-D significantly decreased the transcript levels of *HO-1*, *CAT*, *SOD2*, and *Nrf2*, while significantly increasing *Keap1* transcript levels at 12 and 24 h (*p* < 0.05). And the transcript levels of *GPX1* and *GPX4* were decreased at 24 h (*p* < 0.05). Meanwhile, compared to Gln-D, GLU-D significantly decreased the *HO-1*, *CAT*, and *SOD-2* transcript levels at 12 and 24 h, and *Nrf2* transcript levels were significantly decreased at 12 h (*p* ˂ 0.05). Thus, the above data suggested that Gln-D or GLU-D induced oxidative stress in YRECs, and oxidative damage was more serious in GLU-D treatment than in Gln-D.

### 3.5. Gln-D or GLU-D Induced Inflammatory Response in YRECs

**Figure 5:**
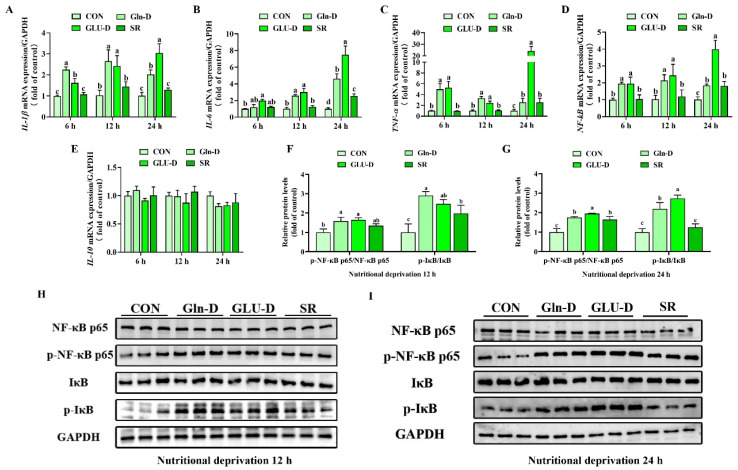
Effects of Gln-D or GLU-D on the inflammation reaction in YRECs. (**A**–**E**) The mRNA levels of *IL-1β*, *IL-6*, *TNF-α*, *NF-κB*, and *IL-10* at 6 h, 12 h, and 24 h. (**F**,**H**) Phosphorylated NF-κB p65 and IκB expression levels at 12 h. (**G**,**I**) Phosphorylated NF-κB p65 and IκB expression levels at 24 h. Immunoblots were captured and quantified using Image J software, and then the normalized values were calculated and presented as ratios of phosphorylated proteins relative to total proteins. Data, expressed as the rate of control cells at each time point, were expressed as means ± SD, n = 3 independent experiments. Different letters indicate significant differences (*p* < 0.05). CON, control; Gln-D, glutamine deprivation; GLU-D, glucose deprivation; SR, serum replacement. IL-1β = interleukin-1β, IL-6 = interleukin-6, TNF-α = tumor necrosis factor-α, NF-κB p65= nuclear factor-κB p65, IL-10 = interleukin-10, p-NF-κB p65 = phospho-NF-κB p65, IκB = inhibitor of NF-κB; p-IκB = phospho-IκB; GAPDH = glyceraldehyde-3-phosphatedehydrogenase.

We next evaluated the influence of nutrient deprivation on inflammatory response in YRECs by q-PCR. As shown in Figure 5A–E, SR did not change the transcript levels of *IL-6*, *IL-1β*, *TNF-α*, and *NF-κB* at 6 and 12 h compared with the control group (*p >* 0.05). However, SR significantly increased the transcript levels of *IL-6*, *TNF-α*, and *NF-κB* at 24 h compared with the control group (*p* < 0.05). Compared with the SR, Gln-D or GLU-D significantly increased the transcript levels of *IL-1β*, *TNF-α*, and *NF-κB* at 6 and 12 h, while increasing the transcript levels of *IL-1β* and *IL-6* at 24 h (*p* < 0.05). In addition, GLU-D significantly increased the transcript levels of *TNF-α* and *NF-κB* at 24 h (*p* < 0.05). Whereas, compared with Gln-D, GLU-D significantly increased the *IL-1β*, *IL-6*, *TNF-α*, and *NF-κB* transcript levels at 24 h (*p* < 0.05). Moreover, Gln-D, GLU-D, or SR had no effect on transcript levels of the anti-inflammatory factor *IL-10* at three time points (*p >* 0.05), but a decreasing trend was found at 24 h (*p* = 0.051). Furthermore, as shown in Figure 5F–I, compared with the control, Western blot analysis showed that the phosphorylation level of IκB at 12 h and the phosphorylation level of NF-κB p65 at 24 h in the SR group were significantly improved (*p* < 0.05). Moreover, compared with the SR, Gln-D significantly increased the phosphorylation level of IκB at 12 and 24 h (*p* < 0.05), and GLU-D significantly improved the levels of p-NF-κB p65 and p-IκB compared to the SR and Gln-D groups at 24 h (*p* < 0.05). Thus, Gln-D or GLU-D induced an inflammation response in YRECs at 12 h and 24 h, and the inflammation response was more serious in the GLU-D group at 24 h.

### 3.6. Gln-D or GLU-D Damaged Tight Junctions in YRECs

**Figure 6:**
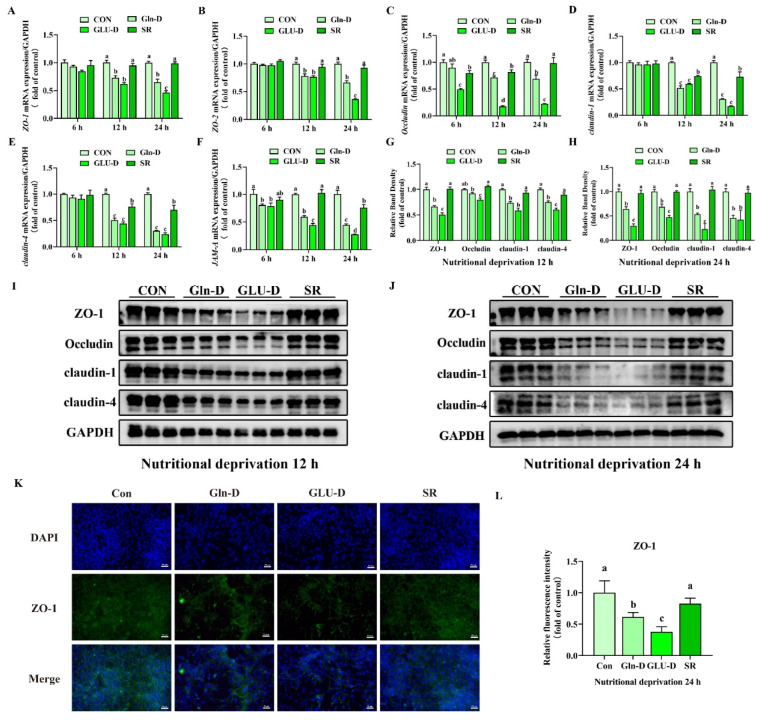
Effects of Gln-D or GLU-D on the tight junction in YRECs. (**A**–**F**) The mRNA expression levels of *ZO-1*, *ZO-2*, *Occludin*, *claudin-1*, *claudin-4*, and *JAM-A* at 6 h, 12 h, and 24 h. (**G**,**I**) The protein expression levels of claudin-1, claudin-4, Occludin, and ZO-1, GAPDH were used as a loading control at 12 h. (**H**,**J**) The protein expression levels of claudin-1, claudin-4, Occludin, and ZO-1, GAPDH were used as a loading control at 24 h. (**K**,**L**) The fluorescence localization of ZO-1 at 24 h (200×). Data, expressed as the rate of control cells at each time point, were expressed as means ± SD, n = 3 independent experiments. Different letters indicate significant differences (*p* < 0.05). CON, control; Gln-D, glutamine deprivation; GLU-D, glucose deprivation; SR, serum replacement. ZO-1 = zonula occludens 1, ZO-2 = zonula occludens 2, JAM-A = junctional adhesion molecule-A; GAPDH = glyceraldehyde-3-phosphatedehydrogenase.

To assess whether nutrient deprivation damaged the tight junction of the YRECs monolayer, we determined the expression of the tight junction-related mRNA and protein. In Figure 6A–F, compared with the control, SR significantly decreased the transcript levels of *Occludin*, *claudin-1*, and *claudin-4* at 12 h but significantly decreased the transcript levels of *claudin-1*, *claudin-4*, and *JAM-A* at 24 h (*p* < 0.05). Compared with the SR, Gln-D or GLU-D significantly decreased the transcript levels of *ZO-1*, *ZO-2*, *Occludin*, *claudin-1*, *claudin-4*, and *JAM-A* at 12 and 24 h (*p* < 0.05). In addition, compared with Gln-D, GLU-D significantly decreased the transcript levels of *ZO-1* and *ZO-2* at 24 h and significantly decreased the transcript levels of *Occludin* and *JAM-A* at 12 h and 24 h (*p* < 0.05). As shown in Figure 6G–J, Gln-D or GLU-D significantly decreased the protein expression levels of ZO-1, Occludin, claudin-1, and claudin-4 at 12 and 24 h compared to SR (*p* < 0.05). Moreover, GLU-D significantly decreased the protein levels of ZO-1, Occludin, and claudin-4 at 12 h and the protein levels of ZO-1, claudin-1, and Occludin at 24 h compared to Gln-D (*p* < 0.05). As shown in Figure 6K–L, the mean fluorescence intensity of ZO-1 trended the same as the protein expression at 24 h. The results demonstrated that Gln-D or GLU-D decreased the tight junction protein expression, and GLU-D had a significant effect on Gln-D.

### 3.7. Gln-D- or GLU-D-Activated MAPK Signaling Pathway in YRECs

**Figure 7:**
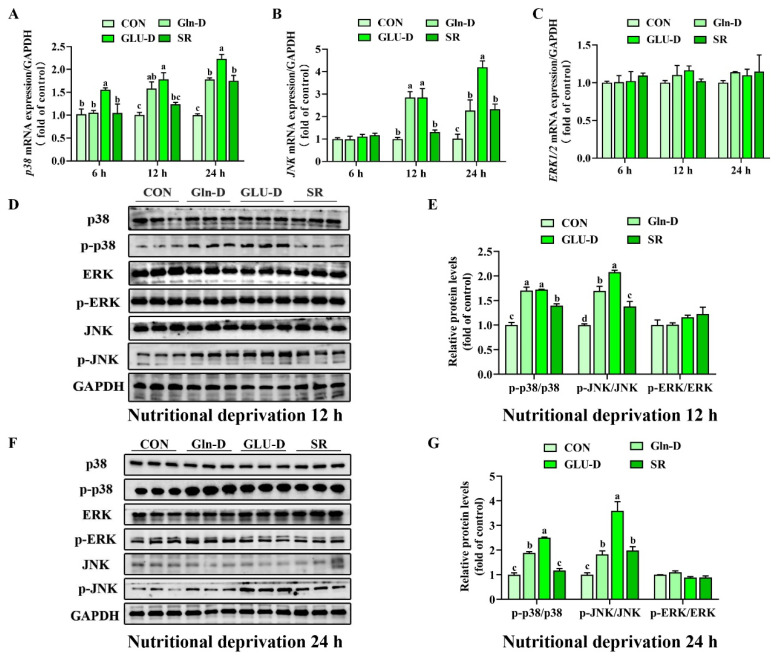
Effects of Gln-D or GLU-D on the MAPK signaling pathway in YRECs. (**A**–**C**) The mRNA expression levels of *p38 MAPK*, *JNK*, and *ERK1/2* at 6 h, 12 h, and 24 h. (**D**,**E**) Phosphorylated p38 MAPK, JNK, and ERK1/2 expression levels at 12 h. (**F**,**G**) Phosphorylated p38 MAPK, JNK, and ERK1/2 expression levels at 24 h. Immunoblots were captured and quantified using Image J software, and then the normalized values were calculated and presented as ratios of phosphorylated proteins relative to total proteins. Data, expressed as the rate of control cells at each time point, were expressed as means ± SD, n = 3 independent experiments. Different letters indicate significant differences (*p* < 0.05). CON, control; Gln-D, glutamine deprivation; GLU-D, glucose deprivation; SR, serum replacement. p38 MAPK = p38 mitogen-activated protein kinase; JNK = c-junN-terminal kinase; ERK1/2 = extracellular signal-regulated 1/2; GAPDH = glyceraldehyde-3-phosphatedehydrogenase.

To demonstrate that nutrient deprivation was involved in the initiation of the MAPK pathway, we determined the mRNA and protein expression of the MAPK signaling molecule. As shown in Figure 7A–C, compared with the control group, SR significantly increased the transcript levels of *p38 MAPK* and *JNK* at 24 h (*p* < 0.05). Compared with the SR, Gln-D significantly improved the *JNK* mRNA level at 12 h (*p* < 0.05), and GLU-D significantly increased the transcript levels of *p38 MAPK* and *JNK* at 12 and 24 h (*p* < 0.05). Compared with Gln-D, GLU-D significantly increased the *p38 MAPK* and *JNK* mRNA expression levels at 24 h (*p* < 0.05). As expected in Figure 7D–G, compared with the control group, SR increased p-JNK at 12 and 24 h (*p* < 0.05). Moreover, Gln-D or GLU-D significantly increased phosphorylation levels of p38 MAPK and JNK at 12 h and p-p38 MAPK at 24 h compared to SR (*p* < 0.05). Furthermore, GLU-D significantly increased the phosphorylation level of JNK at 24 h (*p* < 0.05). In addition, GLU-D significantly increased the phosphorylation level of JNK compared to the Gln-D at 12 and 24 h (*p* < 0.05). The results suggested that Gln-D might damage YRECs through the p38 MAPK/JNK signaling pathways at 12 h, but only through the p38 MAPK signaling at 24 h, GLU-D might damage YRECs through the p38 MAPK/JNK signaling pathway at 12 h and 24 h.

### 3.8. Gln-D or GLU-D Induced Inflammation and Damaged Tight Junction in YRECs Through the p38 MAPK/JNK Signaling Pathway

**Figure 8:**
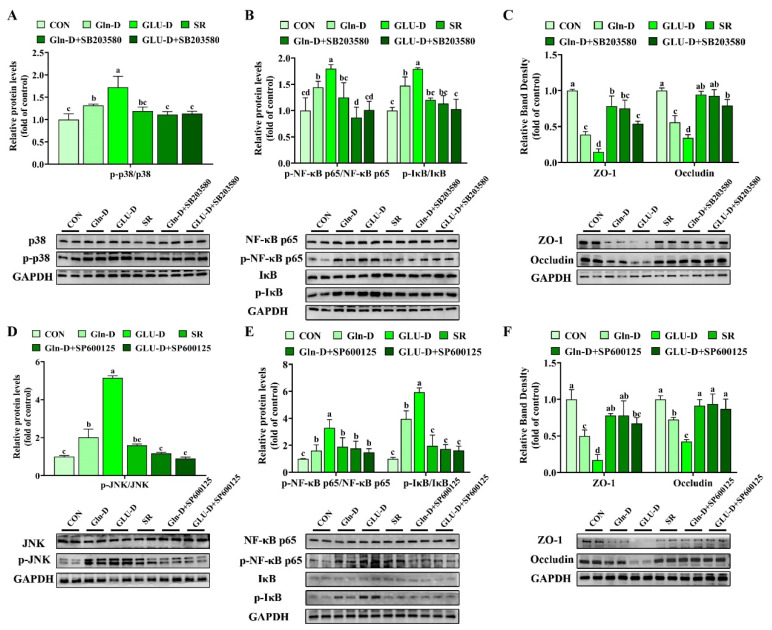
p38 MAPK inhibitor (SB203580) and JNK inhibitor (SP600125) reversed the inflammation and tight junction induced by Gln-D or GLU-D in YRECs at 24 h. The YRECs were pretreated with 10 μM of p38 MAPK inhibitor (SB203580) or JNK inhibitor (SP600125) for 1 h. (**A**–**C**) p38 MAPK inhibitor (SB203580) reversed the inflammation and tight junction induced by Gln-D or GLU-D in YRECs at 24 h. Phosphorylated p38 MAPK, NF-κB p65, and IκB expression levels. The protein expression levels of ZO-1 and Occludin. GAPDH was used as a loading control at 24 h. (**D**–**F**) JNK inhibitor (SP600125) reversed the inflammation and tight junction induced by Gln-D or GLU-D in YRECs at 24 h. Phosphorylated JNK, NF-κB p65, and IκB expression levels. The protein expression levels of ZO-1 and Occludin. GAPDH was used as a loading control at 24 h. Immunoblots were captured and quantified using Image J software, and then the normalized values were calculated and presented as ratios of phosphorylated proteins relative to total proteins. Data, expressed as the percent of control cells, were expressed as means ± SD, n = 4 independent experiments. Different letters indicate significant differences (*p* < 0.05). CON, control; Gln-D, glutamine deprivation; GLU-D, glucose deprivation; SR, serum replacement. p38 MAPK = p38 mitogen-activated protein kinase; JNK = c-junN-terminal kinase; NF-κB p65 = nuclear factor-κB p65; p-NF-κB p65 = phospho-NF-κB p65, IκB = inhibitor of NF-κB; p-IκB = phospho-IκB; ZO-1 = zonula occludens 1; GAPDH = glyceraldehyde-3-phosphatedehydrogenase.

The inhibition of p38 MAPK (SB203580) and JNK (SP600125) on Gln-D or GLU-D induction at 24 h was examined. In Figure 8A–C, the increase in p-p38 MAPK and p-NF-κB p65 proteins following the GLU-D and Gln-D group and p-IκB proteins in the Gln-D group were significantly reduced by SB203580 (*p* < 0.05). Furthermore, the decrease in ZO-1 and Occludin protein levels following Gln-D or GLU-D was significantly increased by SB203580 (*p* < 0.05). As shown in Figure 8D–F, the increase in p-JNK, p-NF-κB p65, and p-IκB following GLU-D was significantly reduced by SP600125 (*p* < 0.05). The decrease in ZO-1 and Occludin protein levels following Gln-D or GLU-D was significantly improved by SP600125 (*p* < 0.05). And the increase in p-JNK and p-IκB following Gln-D was significantly reduced by SP600125 (*p* < 0.05).

**Figure 9:**
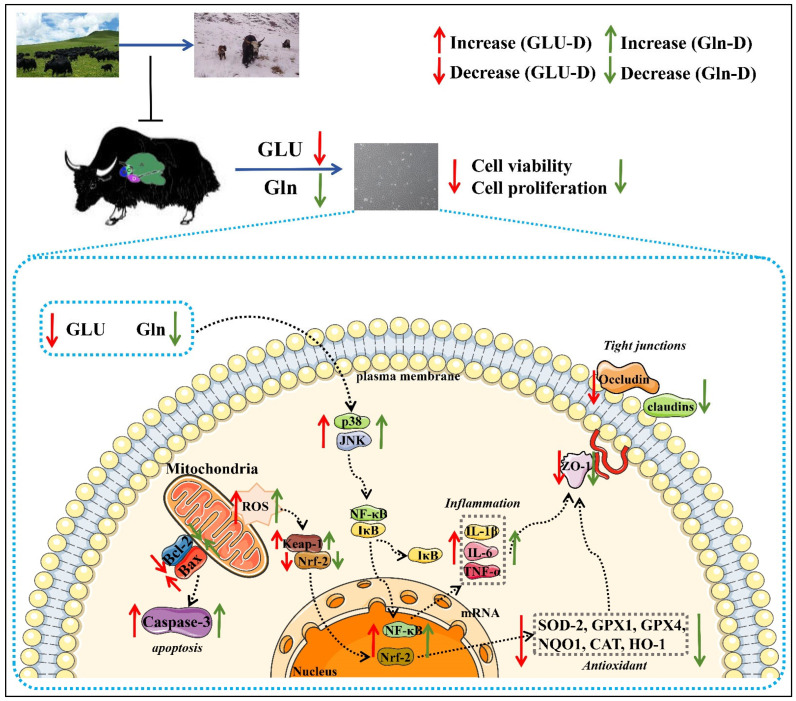
Effects of Gln-D or GLU-D on inflammation and tight junction disruption in yak rumen epithelial cells at 24 h.

To sum up, these results suggested that Gln-D or GLU-D induced inflammation and damaged tight junctions in YRECs through the p38 MAPK/JNK signaling pathway (Figure 9).

## 4. Discussion

As yak live in the plateau regions, nutrient deficiency of yak has become a common phenomenon because of pasture deficiency in the cold season [22,23]. In ruminants, the rumen plays an important function in the digestion and absorption of nutrients. Studies showed that inadequate nutrient intake damaged the gastrointestinal epithelium, which further inhibited gastrointestinal epithelial nutrient uptake and transport, thereby affecting the growth and development of the animal [24,25]. It was reported that insufficient nutrients in the early stage of yak could damage the functional integrity of the rumen epithelial barrier and induce an inflammatory response, which hindered nutrient absorption and reduced growth performance [4]. As is well known, ruminant animals use volatile fatty acids (VFAs) as their important source of energy [26]. However, a study on yaks showed that the GLU level in serum significantly reduced under starvation conditions [5], which might decrease the GLU concentration in rumen epithelium. In addition, a study on rats discovered that starvation significantly reduced the glutamine synthetase activity, thereby decreasing the Gln concentration in intestinal epithelial cells [27]. However, the effects of Gln-D or GLU-D on barrier function in YRECs are still largely unclear. Thus, to fill this gap, we use the YRECs to research the effect of nutrient deficiency on the structural barrier and immune function of yak rumen epithelial and possible molecular mechanisms. Our results indicated that GLU-D and Gln-D induced an apoptotic oxidative damage inflammatory response, and damaged tight junctions in YRECs might be through the p38 MAPK/JNK signaling pathway. Moreover, YRECs were more sensitive to GLU-D than Gln-D.

### 4.1. Gln-D or GLU-D Inhibited the Growth of YRECs

It is first evident that loss of function alters cell morphology [28]. In our results, the morphological changes in the YRECs of the Gln-D and GLU-D groups were observed with HE staining. The SR does not change the cell morphology of YRECs. There were fewer cell–cell contacts, and the cells became spindle-shaped at 12 h of the GLU-D group or 24 h of the Gln-D group. These results suggested that nutrition deprivation lead to cell morphological changes, and YRECs were more sensitive to GLU-D. CCK-8 could be used to assess cell viability since it can show how sensitive live cells are to extrinsic stimulation [29]. Furthermore, cell proliferation determines cell viability [30]. Our results showed that SR did not change cell viability or cell proliferation. Gln-D or GLU-D significantly reduced cell viability and cell proliferation at 12 h and 24 h. Moreover, cells deprived of GLU showed reduced viability and proliferation compared with the Gln. Similarly, another study found that Gln-D or GLU-D decreased the cell viability and cell number in cen3tel cells at 12–20 h [31]. Furthermore, GLU-D inhibited cell proliferation in Met5A cells at 24 h [32]. Cell proliferation and apoptosis are normal physiological processes of cells and maintain homeostasis and normal physiological function of the body [33], while excessive apoptosis is harmful to the body [34]. During apoptosis, caspase-3 is the important executor [35]. Cytochrome c release and caspase activation are inhibited by BCL-2 anti-apoptotic proteins [36]. As expected, we found that Gln-D or GLU-D down-regulated the gene expression of *Bcl-2* and increased the transcript levels of *caspase-3* and *Bax* in YRECs. GLU-D significantly increased the protein levels of Bax and Cleaved-caspase-3 at 12 and 24 h. Moreover, compared with Gln-D, GLU-D significantly increased the transcript levels of *caspase-3* and *Bax* and protein levels of Bax and Cleaved-caspase-3. A similar study found that Gln-D induced apoptosis in MCEC cells [37]. In addition, GLU-D induced apoptosis in HMM cells [32]. These results implied that nutrition deprivation inhibited cell proliferation and induced apoptosis in YRECs. Also, YRECs were more sensitive to cell viability and apoptosis induced by GLU-D than Gln-D.

### 4.2. Gln-D or GLU-D Induced Oxidation Stress in YRECs

Oxidative stress is closely associated with cell damage [38]. The imbalance in ROS generation and scavenging induced by stress disrupted the balance of redox homeostasis in cells, which induced oxidation stress [39,40]. Meanwhile, the increase in intracellular MDA levels is an important indicator of oxidative stress [41]. Antioxidants like GSH-PX, SOD, HO-1, NQO1, and CAT play a crucial function in scavenging the free radicals [42,43]. Compared with the control group, GLU-D increased the ROS level at 24 h. Gln-D or GLU-D increased the MDA level and decreased the GSH-PX and SOD enzyme activities at 24 h. Meanwhile, the MDA and SOD levels between GLU-D and Gln-D reached a significant level. A similar study demonstrated that Gln-D increased the ROS levels in MCF7 cells [11]. Another study found that GLU-D increased the level of ROS and decreased the enzyme activities of GPX1, in turn, inducing oxidative damage in PDA cells [17]. Thus, our results indicated that Gln-D or GLU-D might induce oxidative damage in YRECs. It is reported that the mRNA expression could regulate antioxidant enzyme activity [44]. Therefore, the down-regulated enzyme activity of GSH-PX and total SOD is in accordance with the mRNA expression of GPX1, GPX4 and SOD-2 [45,46]. Furthermore, the Nrf2/Keap1 signaling pathway is critical for the protection of organisms against oxidative damage [47]. Physiologically, in the cytoplasm, Nrf2 is bound to its repressor Keap1 [48]. Translocation of Nrf2 to the nucleus occurs during oxidative stress, where it is released from the Nrf2/Keap1 complex [49]. In the present study, Gln-D or GLU-D significantly decreased *NQO1*, *GPX4*, *GPX1*, *HO-1*, *CAT*, *SOD2*, and *Nrf2* transcript levels and increased *Keap1* transcript levels. Meanwhile, compared with Gln-D, GLU-D significantly decreased the transcript levels of *HO-1*, *CAT*, and *SOD* at 12 h and 24 h. A study found that GLU-D decreased the CAT level in PC12 cells [50]. In another study, Gln-D increased the H_2_O_2_ levels in EC9706 cells and ECa109 cells [51]. Reduced expression of HO-1 and Nrf2 can induce cellular oxidative damage [52,53]. Furthermore, Keap1 serves as an inhibitor of Nrf2 within cytoplasmic [54], so the expression of them showed opposite trends. The study has discovered that oxidative damage increased the *Keap1* mRNA expression and attenuated the Nrf2 expression in mouse tumor tissues [55]. Our results are consistent with the above. Gln-D or GLU-D decreased the *Nrf2* gene expression and increased the *Keap1* gene expression. Thus, our results suggested that Gln-D or GLU-D induced oxidative damage in YRECs. Moreover, compared with Gln-D, GLU-D induced more severe oxidative damage.

### 4.3. Gln-D or GLU-D Induced an Inflammation Reaction in YRECs

Oxidative stress can induce inflammation reactions by promoting the secretion of inflammatory cytokines [56]. IL-6, IL-1β, and TNF-α are common pro-inflammatory biomarkers [57,58]. We found that Gln-D or GLU-D increased the *IL-1β*, *IL-6*, and *TNF-α* transcript levels compared to the SR. Furthermore, GLU-D increased the transcript levels of *IL-6*, *IL-1β*, and *TNF-α* compared to Gln-D at 24 h. Our previous study found that nutrient shortage caused by insufficient forage increased the transcript levels of *IL-6*, *IL-1β*, and *TNF-α* in yak ruminal epithelial tissue [4]. Another in vitro study found that IL-6 secretion was increased by GLU-D in ASC cells [12]. Our results showed that Gln-D or GLU-D induced an inflammation reaction, which was more sensitive in GLU-D. Inflammation-related genes are regulated by NF-κB, which is a major nuclear transcription factor [59]. In addition, a greater level of IκB suggests that NF-κB is located in the cytoplasm, whereas a higher level of p-IκB suggests that NF-κB is located in the nucleus [58]. In our results, we found that Gln-D increased the IκB phosphorylation level at 24 h compared to the SR group. GLU-D improved the protein levels of p-IκB and p-NF-κB p65 at 24 h compared to the SR and Gln-D groups. Further evidence suggested that Gln-D or GLU-D induced inflammation reaction might be through the NF-κB p65 signaling pathway in YRECs. Moreover, compared with Gln-D, GLU-D induced a more severe inflammation reaction.

### 4.4. Gln-D or GLU-D Damaged Tight Junctions in YRECs

Research has already demonstrated that the imbalance between cell proliferation and apoptosis could damage epithelial barriers [60]. Furthermore, oxidative stress might disrupt epithelial barrier function by disturbing the tight junction [56]. Epithelial barrier integrity is maintained by tight junction proteins [61]. Our results found that Gln-D or GLU-D reduced the transcript levels of *ZO-1*, *ZO-2*, *JAM-A*, *claudin-1*, *claudin-4*, and *Occludin*, as well as decreased the protein expression levels of ZO-1, claudin-1, claudin-4, and Occludin in YRECs. Between Gln-D and GLU-D, a significant level was reached. A similar study found that GLU-D decreased ZO-1 protein expression in Bend.3 cells [62]. The results demonstrated that nutrient shortage disrupted the tight junctions. GLU-D had more severe damage than Gln-D.

### 4.5. Gln-D or GLU-D Activated the MAPK Signaling Pathway in YRECs

The MAPK signaling pathway is instrumental in regulating various cellular functions, including cell proliferation, apoptosis, oxidative stress, and inflammation [13]. It was reported that the p38 MAPK signaling pathway could mediate the exogenous stimulus-induced tight junction dysregulation in distal renal tubular epithelial cells [18]. Moreover, our previous study found that nutrient deficiency increased the protein abundance of p38 MAPK, ERK1/2, and JNK in yak rumen epithelial tissue. Thus, we hypothesize that the MAPK pathway exerts an important function in cellular damage induced by Gln-D or GLU-D in YRECs. To verify this hypothesis, *p38 MAPK*, *JNK*, and *ERK1/2 MAPK* transcript levels or phosphorylation levels were examined by q-PCR and WB. Our data demonstrated that Gln-D increased the transcript levels of *JNK* and phosphorylation level of JNK at 12 h and increased the level of p-p38 MAPK at 24 h compared to the SR. GLU-D up-regulated the transcript levels of *JNK* and *p38 MAPK* and increased the protein levels of JNK and p38 MAPK at 24 h compared to the SR and Gln-D groups. The data suggested that Gln-D damaged YRECs might be through the JNK signaling pathway at 12 h of the p38 MAPK signaling pathway at 24 h. Also, GLU-D damaged YRECs might be through the JNK signaling pathway at 12 h and the p38 MAPK/JNK signaling pathway at 24 h. A similar result showed that GLU-D activated p38 MAPK in N2A cells [20] and Gln-D activated JNK in U2OS cells [19]. To verify these results, we used inhibitors SB203580 and SP600125 of p38 MAPK and JNK to pretreat YRECs before Gln-D and GLU-D. Our results proved that SB203580 and SP600125 significantly alleviated GLU-D-induced inflammation and tight junction damage at 24 h. SP600125 significantly alleviated Gln-D-induced inflammation and tight junction damage at 24 h. These data indicated that Gln-D and GLU-D damaged YRECs through JNK and p38 MAPK/JNK signaling pathways at 24 h, respectively.

It is well known that short-chain fatty acids (SCFA) are an important energy source for rumen epithelium cells. However, in our study, we did not consider the influence of SCFA on YRECs. In addition, we did not consider the dose responses and the interchangeability of substrates (GLU, Gln, and SCFA), and we applied extremely harsh experimental conditions with glucose or glutamine being either present or completely absent. Therefore, in subsequent experiments, we will more fully explore the dose responses and the interchangeability of substrates (GLU, Gln, and SCFA).

## 5. Conclusions

In summary, our data indicated that Gln-D beyond 12 h disrupted YREC cellular tight junctions by inducing oxidative stress, and GLU-D beyond 12 h disrupted YREC cellular tight junctions by inducing apoptosis and oxidative stress. Interestingly, GLU-D-induced damage was more severe than Gln-D at 24 h. The damage induced by Gln-D or GLU-D might be associated with the activation of p38 MAPK and JNK signaling pathways in YRECs. GLU-D disrupted the tight junctions through p38 MAPK and JNK signaling pathways compared to Gln-D in YRECs. Our study provided molecular mechanisms for forage deficiency-induced impairment of yak rumen epithelium.

## Data Availability

The original contributions presented in the study are included in the article/Supplementary Materials, further inquiries can be directed to the corresponding authors.

## Supplementary Materials

The following supporting information can be downloaded at: https://www.mdpi.com/article/10.3390/ani14223232/s1, Table S1: The information of antibodies (Western blot). Table S2: Real-time polymerase chain reaction primer sequences. Figure S1: Image of the full gel.

## Abbreviations

Gln, glutamine; GLU, glucose; YRECs, yak rumen epithelial cells; ZO-1, zonula occludens-1; JAM-A, junctional adhesin molecule-A; MAPK, mitogen-activated protein kinase; Gln-D, glutamine deprivation; GLU-D, glucose deprivation.
