# Supplementary material for: Effects of Glutamine or Glucose Deprivation on Inflammation and Tight Junction Disruption in Yak Rumen Epithelial Cells
Source: Animals (Basel). 2024 Nov 12;14(22):3232. doi: 10.3390/ani14223232 (PMC11591495; doi:10.3390/ani14223232)
Supplement: Supplementary file 1 [file animals-14-03232-s001.zip › animals-3254957-supplementary.pdf]

Effects of Glutamine or Glucose Deprivation on Inflammation and Tight Junction Disruption in Yak Rumen Epithelial Cells

Supplementary materials

Supplementary Table S1 The information of antibodies (Western blot).

Indices	Host	Source	Catalog No.	Dilution for WB
GAPDH	Rabbit	ABclonal (Wuhan, Hubei, China)	AC001	1:5000
Bcl-2	Rabbit	ABclonal (Wuhan, Hubei, China)	A0208	1:1000
Bax	Rabbit	ABclonal (Wuhan, Hubei, China)	A12009	1:1000
Cleaved-caspsase-3	Rabbit	ABclonal (Wuhan, Hubei, China)	A11040	1:1000
NF-κB p65	Rabbit	ABclonal (Wuhan, Hubei, China)	A11202	1:1000
p-NF-κB p65	Rabbit	ABclonal (Wuhan, Hubei, China)	AP0123	1:1000
IκB	Rabbit	ABclonal (Wuhan, Hubei, China)	A19714	1:1000
p-IκB	Rabbit	ABclonal (Wuhan, Hubei, China)	AP0707	1:1000
claduin-1	Rabbit	Affinity (Golden, Colorado, USA)	DF6919	1:1000
claudin-4	Rabbit	ABclonal (Wuhan, Hubei, China)	A12912	1:1000
Occludin	Rabbit	ABclonal (Wuhan, Hubei, China)	A12621	1:1000
ZO-1	Rabbit	ABclonal (Wuhan, Hubei, China)	A0659	1:1000
p38 MAPK	Rabbit	ABclonal (Wuhan, Hubei, China)	A10832	1:1000
p-p38 MAPK	Rabbit	ABclonal (Wuhan, Hubei, China)	AP0057	1:1000
JNK	Rabbit	Affinity (Golden, Colorado, USA)	AF6318	1:1000
p-JNK	Rabbit	Affinity (Golden, Colorado, USA)	AF3318	1:1000
ERK 1/2	Rabbit	ABclonal (Wuhan, Hubei, China)	A16686	1:1000
p-ERK 1/2	Rabbit	ABclonal (Wuhan, Hubei, China)	AP1120	1:1000

GAPDH: glyceraldehyde-3-phosphatedehydrogenase; **Bcl-2**: B-cell lymphoma 2; **Bax**: B-cell lymphoma 2-associated X protein; **NF-κB p65**: nuclear factor-κB p65; **IκB**: inhibitor of NF-κB; **ZO-1**: zonula occludens 1; **p38 MAPK**: p38 mitogen-activated protein kinase; **JNK**: c-junN-terminal kinase; **ERK1/2**: extracellular signal-regulated 1/2.

Supplementary Table S2 Real-time polymerase chain reaction primer sequences [1]

Gene	Primer sequence (5'-3')	Length, bp	Accession number
GAPDH	F: CGGCACAGTCAAGGCAGAGAAC	116	XM_014482068.1
	R: CCACATACTCAGCACCAGCATCAC		
caspase-1	F: GGATGGGATCTGCGGGACTATG	96	XM_005887200.2
	R: CTGAGGCAATTACGGTTGTTGAATG		
caspase-3	F: GAACCAATGGACCCGTCAATCTG	148	NM_001077840.1
	R: GCACCACTGTCTGTCTCAATACC		
Bax	F: TGCTTCAGGGTTTCATCCAGGG	328	NM_173894.1
	R: GTCCTGATCAACTCGGGCAC		
Bcl2	F: ATGTGTGTGGAGAGCGTCAA	190	NM_001166486.1
	R: GTGCCTTCAGAGACAGCCAG		
NQO1	F: CTCTGGCCAATTCAGAGTGG	111	NM_001034535.1
	R: CAGGATCTGAACTCGGGCAT		
GPX4	F: TCGCAATGAGGCAAGACTGACG	377	NM_001346430.1
	R: TAGCACGGCAGGTCCTTCTCTATC		
GPX1	F: CATCCGCTCTTCGCCTTCCTTC	203	NM_174076.3
	R: GGCTCGATGTCGATGGTCAGAAAG		
HO-1	F: GGCAGCAAGGTGCAAGA	221	NM_001014912.1
	R: GAAGGAAGCCAGCCAAGAG		
CAT	F: TCACTCAGGTGCGGACTTTC	66	NM_001035386.2
	R: TCTCACACAGGCGTTTCCTC		
SOD-2	F: GGGTTGGCTCGGCTTCAATAAGG	208	NM_201527.2
	R: TCGTGCAGTTACATTCTCCCAGTTG		
Nrf2	F: CCCAGTCTTCACTGCTCCTC	165	NM_001011678.2
	R: TCAGCCAGCTTGTCATTTTG		
Keap1	F: GATCTACGTTCTTGGGGGCT	105	NM_001101142.1
	R: CCAGAGGTCATTCGGGTCAC		
IL-1β	F: CTCCGACGAGTTTCTGTGTGACG	120	NM_174093.1
	R: GAGAGGAGGTGGAGAGCCTTCAG		
IL-6	F: CACTGACCTGCTGGAGAAGATGC	115	XM_005901249.2
	R: CCGAATAGCTCTCAGGCTGAACTG		
TNF-α	F: TGAAGGAAGAGGAGAGGCTCATCG	107	XM_005904178.1
	R: GTGGTCATCGGAGTTGCTGGTG		
NF-κB	F: GCCTGCTGAATGCTCTGTCTGAC	143	XM_005887214.2
	R: CTCTGTTTCCTGTTCCACCGACTG		
IL-10	F: GAACCACGGGCCTGACATCAAG	127	XM_005891650
	R: CTTCTCCACCGCCTTGCTCTTG		
ZO-1	F: CCGAATGAAACCGCACACAAACC	107	XM_014476599.1
	R: GTCTCCACGCCACTGTCAAACTC		
ZO-2	F: AACCTCAATTCAGCCAACGACAG	112	XM_005897468.2
	R: CATCATCCATCCCTTCCATCTTTCC		
Occludin	F: GCCTGTGTTGCCTCCACTCTTG	143	XM_005889348.2
	R: CCATAGCCATAACCGTAGCCATAGC		

claudin-1	F: CCCGTGCCTTGATGGTGATTGG<br>R: CATCTTCTGTGCCTCGTCGTCTTC	110	XM_005897671.2
claudin-4	F: TCATCGGCAGCAACATCGTCAC<br>R: CAGCAGCGAGTCGTACACCTTG	110	XM_005892850.2
JAM-A	F: GTGCCTCCATCCAAGCCTACAATC<br>R: GGCATCTCTACTCCATCCTTGAACC	134	XM_010802736.3
p38 MAPK	F: TGCTGGAGAAGATGCTTGTATTGG<br>R: TCGTCGTCAGGATCGTGGTAC	94	XM_005890506.2
JNK	F: CAGAAGCAAGCGTGACAGCA<br>R: TTCCTTGGGCTCCTGAACCT	130	XM_005900620.1
ERK1/2	F: AGGTGTGGTGTTCAAGGTCTCC<br>R: TGATCTCGCCGTCGCTGTAG	100	XM_005893283.2

[1]***NQO1***: NAD(P)H dehydrogenase quinone 1; ***GPX4***: Glutathione peroxidase 4; ***GPX1***: Glutathione peroxidase 1; ***HO-1***: Heme oxygenase 1; ***CAT***: Catalase; ***SOD-2***: Superoxide dismutase 2; ***Nrf2***: Nuclear factor-erythroid 2-related factor 2; ***Keap1***: Kelch-like-ECH-associated protein 1; ***IL-1β***: Interleukin-1β; ***IL-6***: Interleukin-6; ***TNF-α***: Tumor necrosis factor-alpha; ***IL-10***: Interleukin-10; ***ZO-2***: Zonula occludens-2; ***JAM-A***: Junction adhesion molecule-A.

Supplementary Figure S1: Image of the full gel.

Fig.3. (D) 12 h

Bcl-2 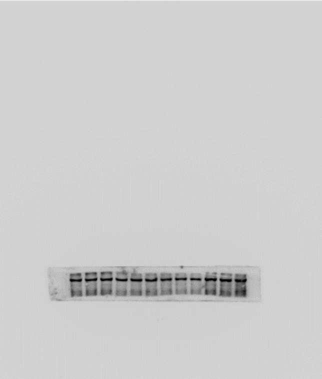 Bax 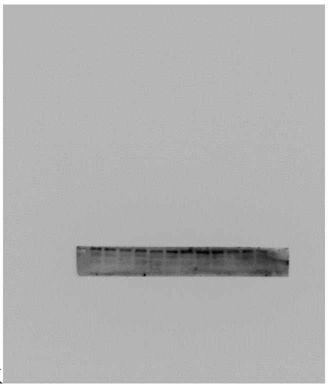

Cleaved-caspase-3 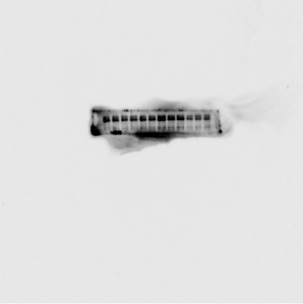 GAPDH 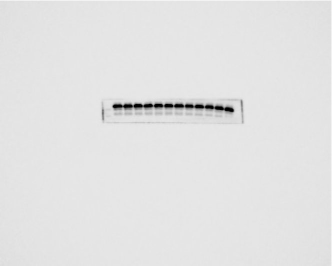

(F) 24 h

Bcl-2 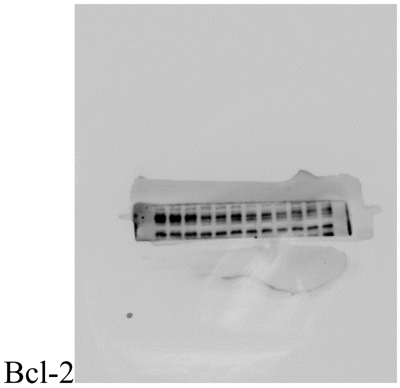 Bax 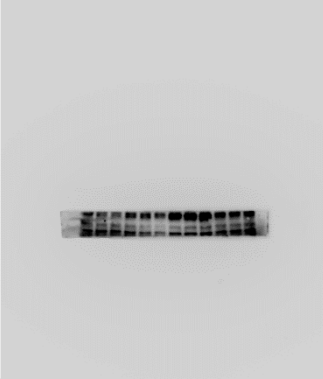

Cleaved-caspase-3 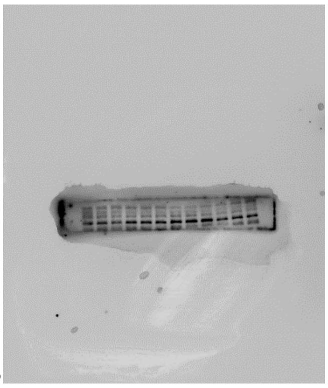 GAPDH 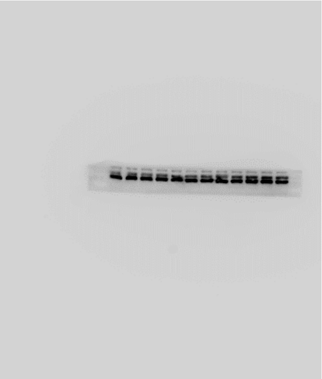

Fig.5. (H) 12 h

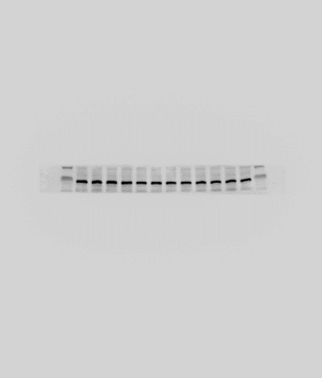

NF-κB p65

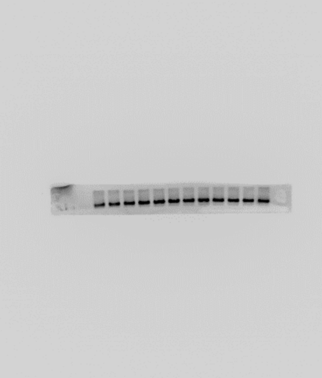

p-NF-κB p65

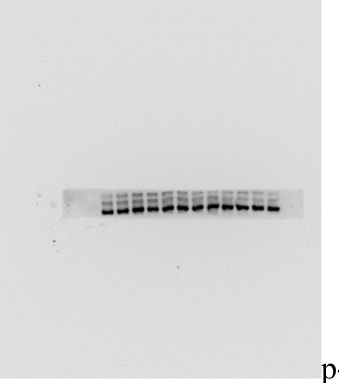

IκB

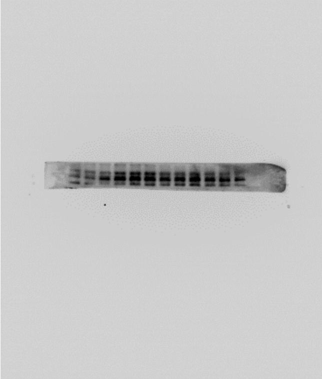

p-IκB

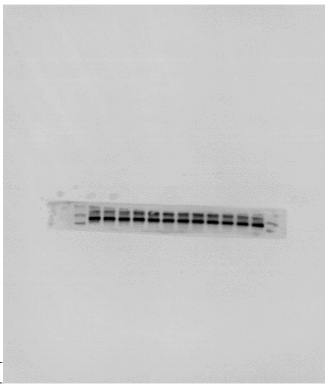

GAPDH

(I) 24 h

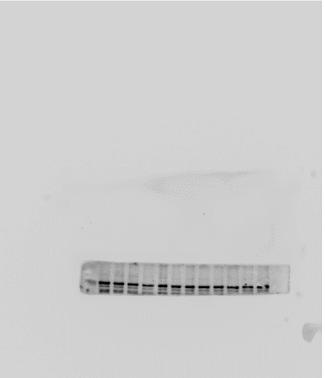

NF-κB p65

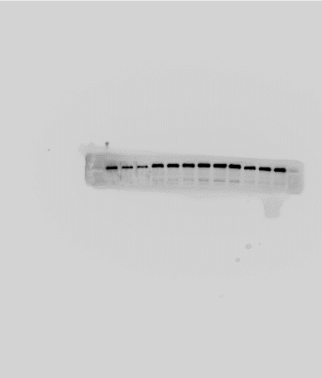

p-NF-κB p65

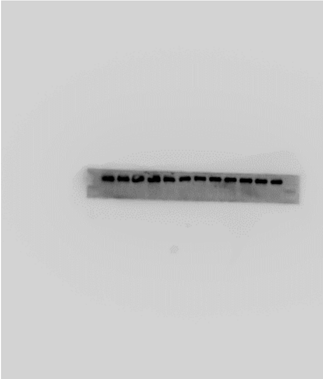
IκB

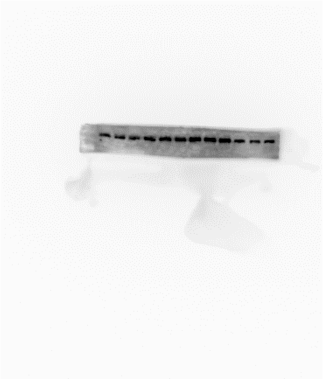
p-IκB

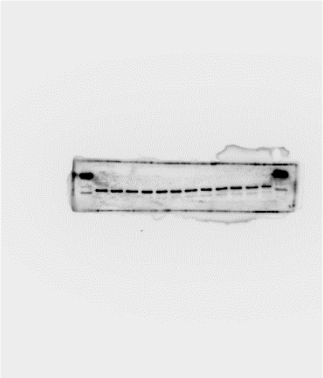
GAPDH

Fig.6. (I) 12 h

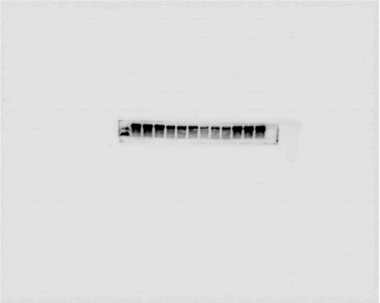
ZO-1

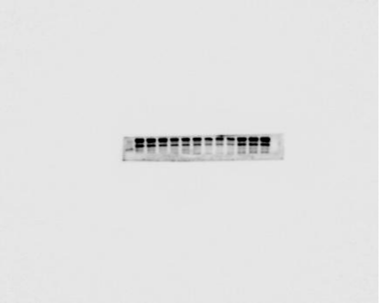
Occludin

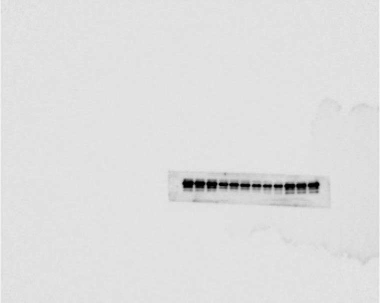
claudin-1

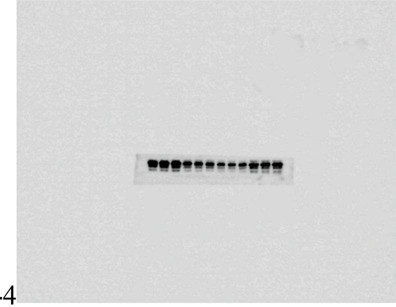
claudin-4

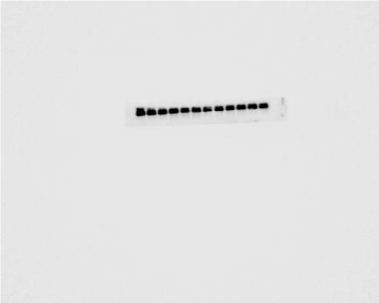
GAPDH

(J) 24 h

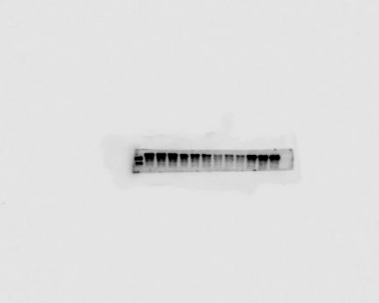
ZO-1

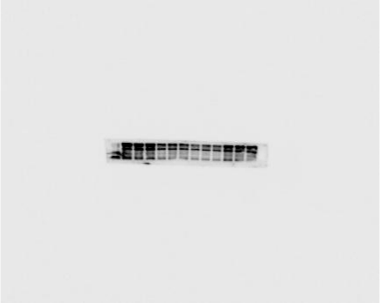
Occludin

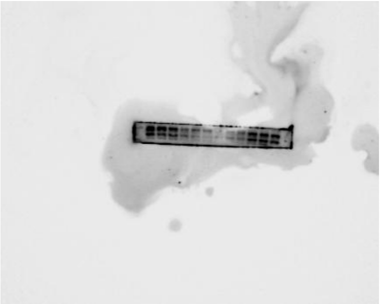
claudin-1

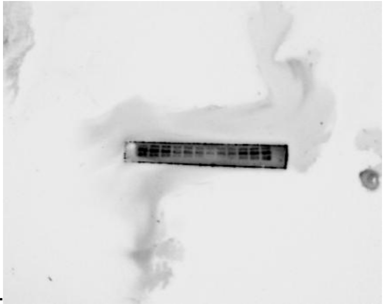
claudin-4

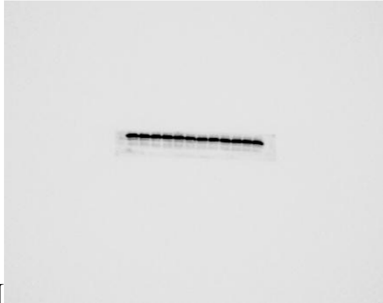
GAPDH

Fig.7. (D) 12 h

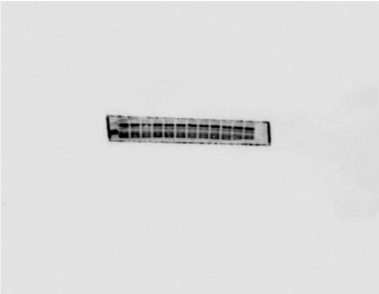
p38 MAPK

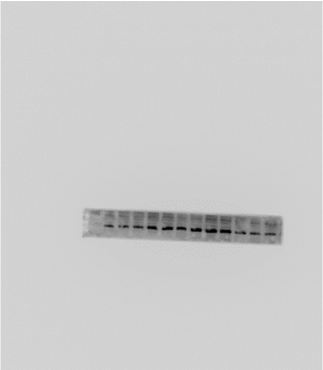
p-p38 MAPK

ERK 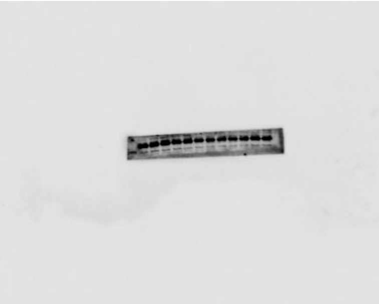 p-ERK 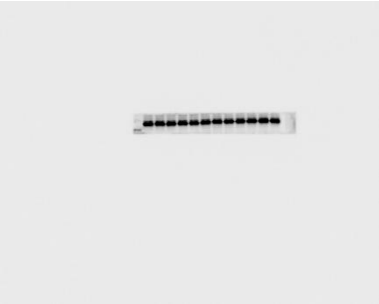

JNK 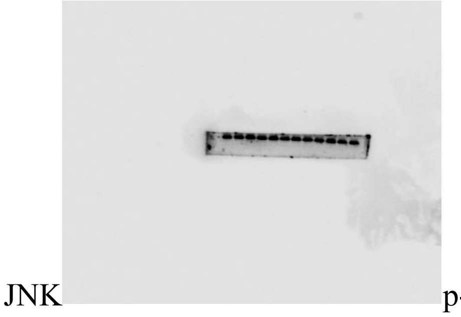 p-JNK 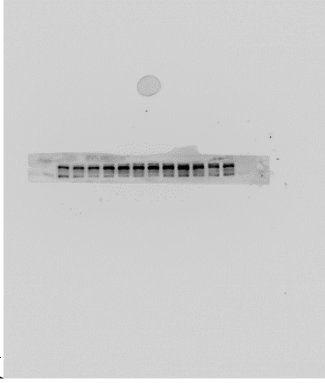

GAPDH 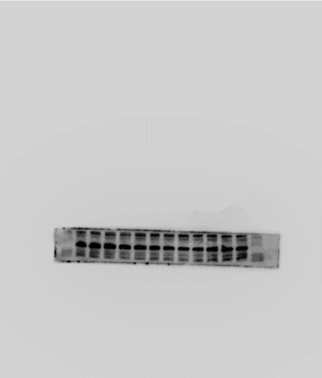

(F) 24 h.

p38 MAPK 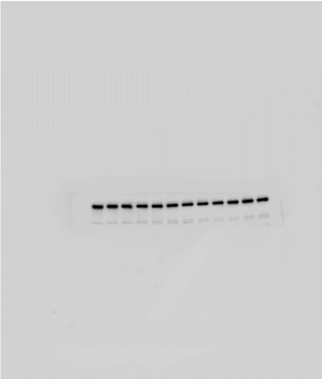 p-p38 MAPK 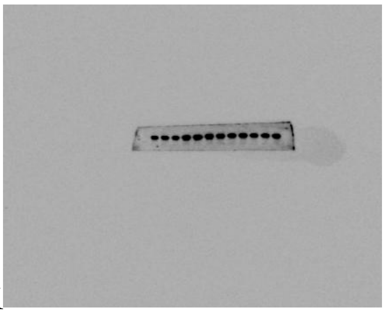

ERK
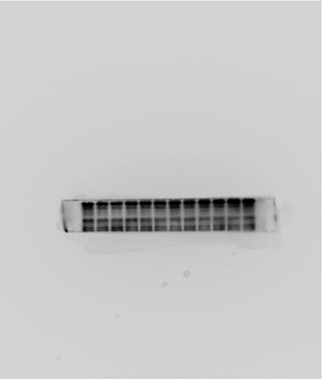
p-ERK
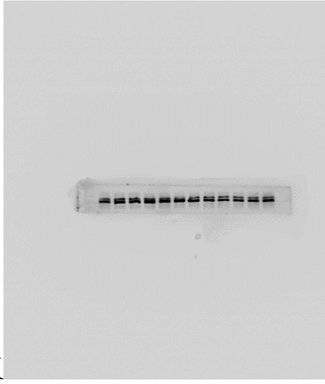
JNK
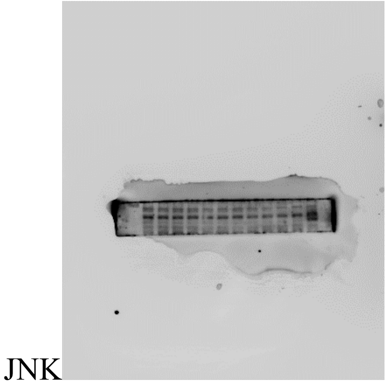
p-JNK
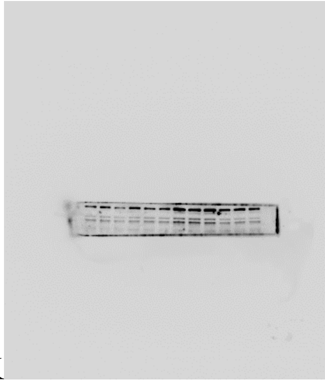
GAPDH
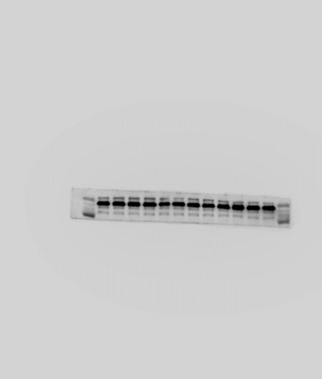

Fig.8. (A)

p38-MAPK
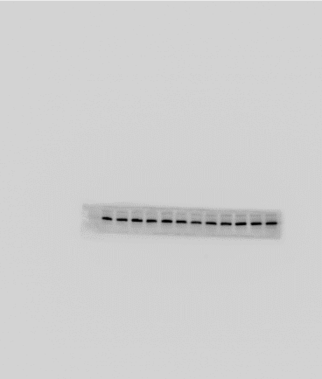
p-p38 MAPK
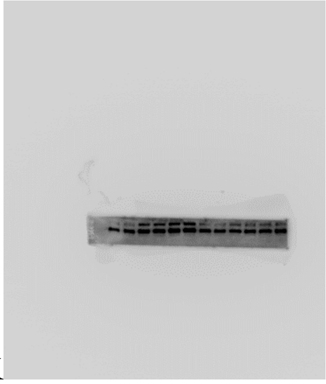

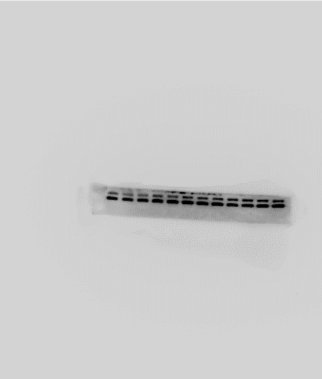

GAPDH

(B)

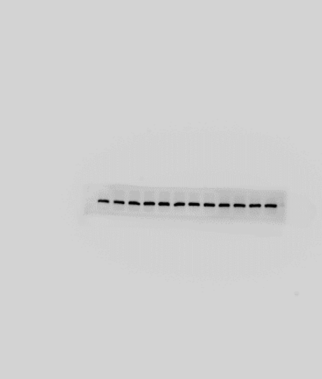

NF-κB p65

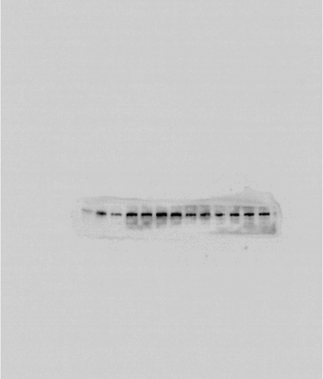

p-NF-κB p65

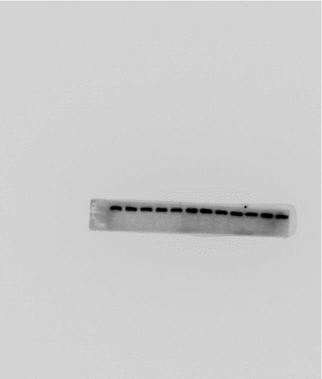

IκB

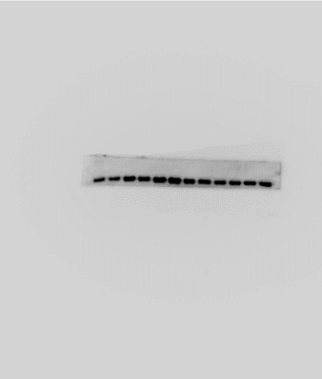

p-IκB

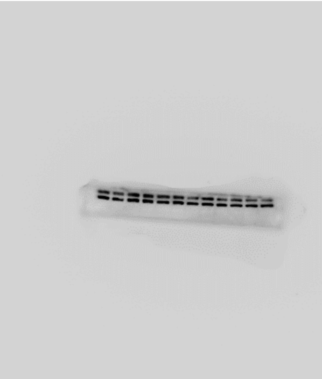

GAPDH

(C)

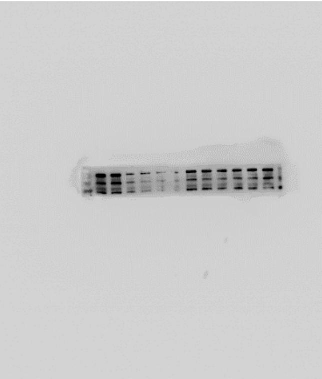

ZO-1

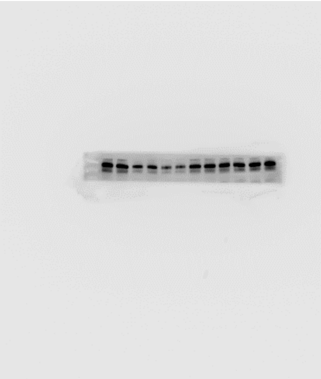

Occludin

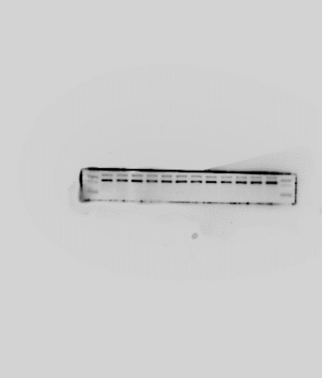

GAPDH

(D)

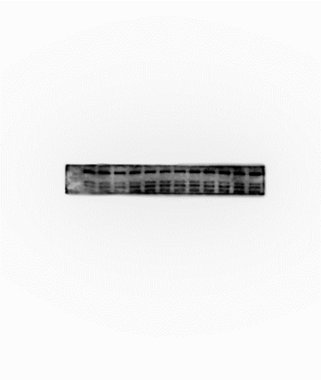

JNK

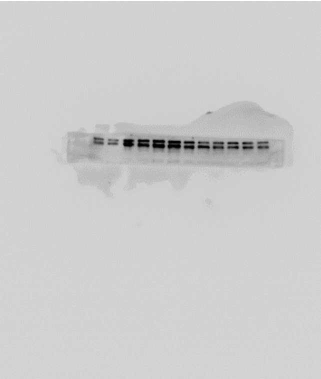

p-JNK

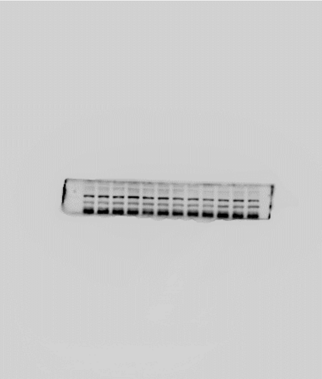

GAPDH

(E)

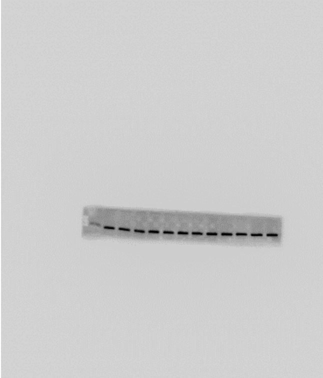

NF-κB p65

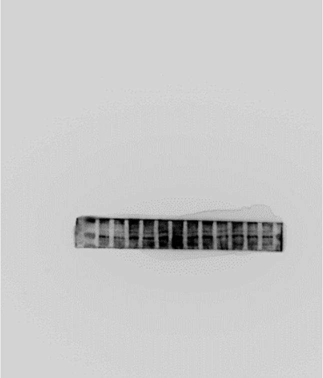

p-NF-κB p65

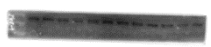

IκB

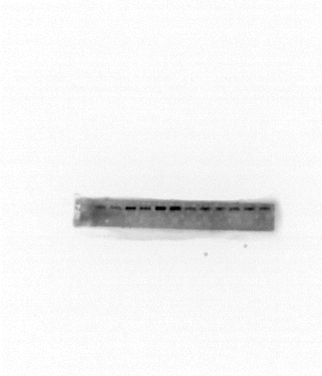

p-IκB

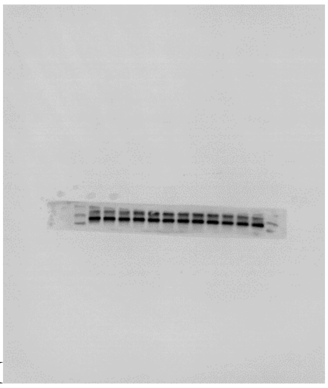

GAPDH

(F)

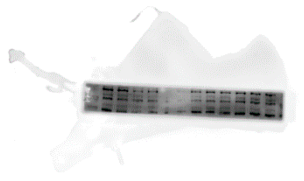

ZO-1

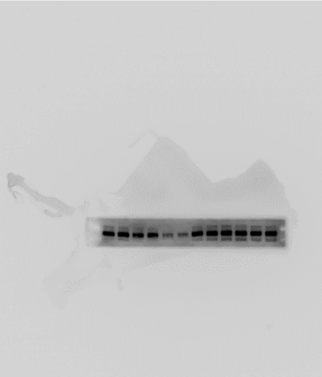

Occludin

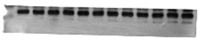

GAPDH
